# Association Between Body Roundness Index and Periodontitis in Adults With Hypertension: A Cross‐Sectional Study

**DOI:** 10.1002/cre2.70429

**Published:** 2026-08-13

**Authors:** Liting Xie, Xiaobin Zhang, Zhiqiang Xu, Zhiyong Pan

**Affiliations:** ^1^ Stomatological Center Affiliated Hospital of Putian University Putian China; ^2^ Department of Otolaryngology Affiliated Hospital of Putian University Putian China

**Keywords:** body roundness index, hypertension, NHANES, periodontitis

## Abstract

**Objectives:**

The association between body roundness index (BRI) and periodontitis among US adults with hypertension remains unclear. This study aimed to investigate the relationship between BRI and periodontitis in this population.

**Material and Methods:**

This cross‐sectional study included 4117 adults with hypertension from the National Health and Nutrition Examination Survey (NHANES) 2009–2014. Multivariable logistic regression models were used to assess the association between BRI and periodontitis. Generalized additive models and two‐piecewise regression models were applied to explore potential nonlinearity and threshold effects. Stratified analyses across covariate‐defined subgroups and sensitivity analyses were conducted to evaluate the robustness of the findings.

**Results:**

After adjustment for covariates, BRI was significantly associated with periodontitis. When BRI was analyzed by quartiles, participants in the highest quartile had significantly higher odds of periodontitis than those in the lowest quartile in both Model I (OR = 1.27, 95% CI: 1.05–1.54, *p* = 0.0122) and Model II (OR = 1.25, 95% CI: 1.03–1.53, *p* = 0.0258). A nonlinear association was observed between BRI and periodontitis. The log‐likelihood ratio test was statistically significant (*p* < 0.05), and the inflection point was estimated to be approximately 6. At or above this point, the OR for the association between BRI and periodontitis was 1.09 (95% CI: 1.04–1.15, *p* = 0.0006), whereas below this point, the corresponding OR was 0.94 (95% CI: 0.87–1.02, *p* = 0.1209). Sensitivity analyses yielded results that were broadly consistent with the primary findings.

**Conclusions:**

Among US adults with hypertension, BRI was associated with periodontitis, and the association was nonlinear.

**Abbreviations**
AAP: American Academy of Periodontology
AL: attachment level
BMI: body mass index
BRI: body roundness index
CDC: Centers for Disease Control and Prevention
CI: confidence interval
FI: Frailty index
FMPE: Full Mouth Periodontal Examination
NCHS: National Center for Health Statistics
NHANES: National Health and Nutrition Examination Survey
OR: odds ratio
PD: probing depth
PIR: poverty income ratio
WC: waist circumference

## 1 Introduction

Periodontitis is a common chronic inflammatory disease of the tooth‐supporting tissues, initiated by dysbiotic plaque biofilms and characterized by progressive destruction of the periodontal attachment apparatus (Glickman 1971; Zhang, Liu et al. 2024). It is a major cause of tooth loss in adults and imposes substantial functional, psychological, social, and economic burdens (Botelho et al. 2022; Chen et al. 2021). In addition to its oral consequences, periodontitis has been associated with several systemic conditions, including cardiovascular disease (Priyamvara et al. 2020), diabetes (Michalowicz et al. 2024), and liver disease (Albuquerque‐Souza and Sahingur 2022). Among these conditions, the association between periodontitis and hypertension has received increasing attention (Del Pinto et al. 2020; Muñoz Aguilera et al. 2020). Accordingly, the present study focused specifically on adults with hypertension, a clinically important high‐risk population. In this context, identifying factors associated with periodontitis in adults with hypertension may be of particular clinical relevance.

Obesity has also been associated with periodontitis in epidemiological studies (Kim et al. 2022). Chronic systemic inflammation has been proposed as one of several plausible mechanisms linking obesity and periodontal disease (Reytor‐González et al. 2024). However, obesity is a heterogeneous condition. Conventional anthropometric indices, such as body mass index (BMI) and waist circumference (WC), are widely used to assess obesity‐related health risks (Kivimäki et al. 2022; Rubino et al. 2025). Nevertheless, BMI cannot distinguish fat mass from lean mass and does not adequately reflect body fat distribution (Gonzalez et al. 2017). Although WC is useful for assessing abdominal obesity, it does not account for height and may incompletely capture body shape and central adiposity (Forero Torres and Forero 2023). The body roundness index (BRI), derived from height and WC, has been proposed as a practical anthropometric measure that may better reflect body fat and visceral adiposity than BMI or WC alone (Thomas et al. 2013).

Most previous studies evaluating obesity in relation to periodontal health have focused on BMI or WC (Chen, Zeng et al. 2025; Liu et al. 2024), whereas evidence regarding BRI remains limited. Although several studies have reported that higher BRI is associated with periodontitis (Xu and Fu 2025; Zhang et al. 2025), data specifically among adults with hypertension are scarce. This population is of particular interest because hypertension commonly coexists with central adiposity (Liu et al. 2022), metabolic abnormalities (Eriksson et al. 1992), and chronic low‐grade inflammation (Zhang et al. 2022), factors that may also be relevant to periodontitis risk. Against this background, the present study aimed to investigate the association between BRI and periodontitis among US adults with hypertension using NHANES 2009–2014 data and to assess whether this association was nonlinear.

## 2 Methods

### 2.1 Study Population

The NHANES is a nationwide cross‐sectional study conducted by the National Center for Health Statistics (NCHS), designed to assess the health and nutritional status of the noninstitutionalized US population. All participants provided informed consent, and all data are publicly available through the NCHS website (https://www.cdc.gov/nchs/nhanes/).

**Figure 1:**
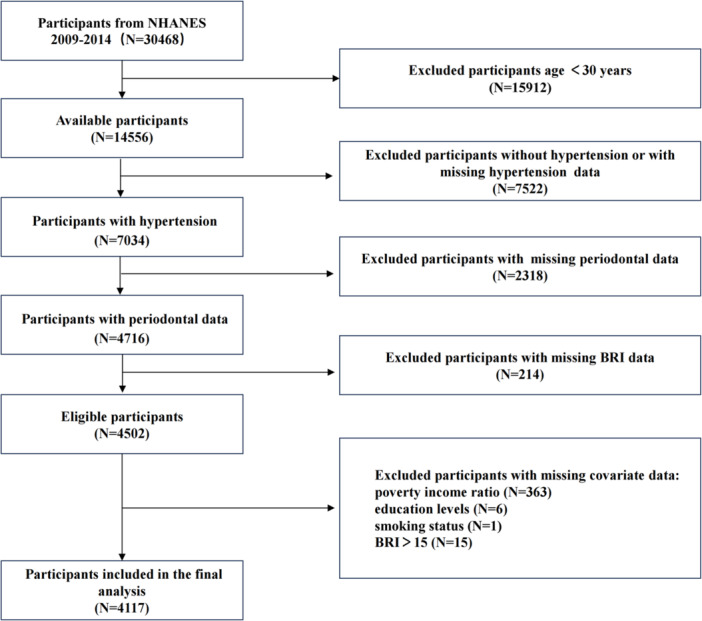
Flowchart of procedures for participants selection and inclusion. BRI, body roundness index; NHANES, National Health and Nutrition Examination Survey.

For this study, we utilized NHANES data from 2009 to 2014, during which the Full Mouth Periodontal Examination (FMPE) data were available. A total of 30,468 participants were initially considered. The following exclusion criteria were applied: participants younger than 30 years were excluded (*N* = 15,912), as the FMPE was only performed in adults aged 30 years and older; those without hypertension or with missing hypertension data (*N* = 7522); those with missing periodontal data (*N* = 2318); those with missing BRI data (*N* = 214); and those with missing data on covariates included in the analysis (*N* = 385). Ultimately, 4117 participants were included in the final analysis. A flowchart of participant selection is shown in Figure 1.

### 2.2 Definition of Variables

#### 2.2.1 BRI

BRI is an anthropometric indicator that integrates height and WC to assess body shape and fat distribution. Anthropometric data, including height and WC, were measured by trained technicians at mobile examination centers following standardized protocols to ensure accuracy and comparability across participants. BRI was subsequently calculated using the established formula (Ding et al. 2024; Zhang, Ma et al. 2024):

$$\text{BRI}\;=364.2-365.5\times \sqrt{1-{\left(\frac{\mathrm{WC}}{2\pi }\right)}^{2}/{\left(0.5\times \mathrm{height}\right)}^{2}}$$

This standardized measurement and calculation approach ensures the reliability and consistency of the anthropometric data used for BRI estimation.

#### 2.2.2 Periodontal Status

Periodontal examinations were obtained from the Oral Health‐Periodontal Examination component of NHANES 2009–2014. Examinations were conducted by trained and calibrated dental hygienists using standardized CDC/AAP protocols. Periodontal status was assessed using probing depth (PD) and attachment level (AL) measured at six sites per tooth. Periodontitis was defined according to the CDC/AAP case definitions (Eke et al. 2012). Mild periodontitis was defined as 2 interproximal sites with AL ≥ 3 mm, and ≥ 2 interproximal sites with PD ≥ 4 mm (not on the same tooth) or one site with PD ≥ 5 mm. Moderate periodontitis was defined as 2 interproximal sites with AL ≥ 4 mm (not on the same tooth), or ≥ 2 interproximal sites with PD ≥ 5 mm (not on the same tooth). Severe periodontitis was defined as 2 interproximal sites with AL ≥ 6 mm (not on the same tooth) and ≥ 1 interproximal site with PD ≥ 5 mm. Participants who did not meet diagnostic criteria for periodontitis were classified as having no periodontitis. For analysis, participants were categorized into two groups: no periodontitis and periodontitis; the periodontitis group included those with mild, moderate, or severe periodontitis.

#### 2.2.3 Definition of Hypertension

Blood pressure was measured at the mobile examination center by trained examiners according to standardized NHANES protocols. Before measurement, participants were asked to remain seated and rest quietly for 5 min. Using a mercury sphygmomanometer, an appropriately sized cuff was applied to the right arm, which was exposed. Three consecutive blood pressure readings were obtained for each participant. If a reading was interrupted or deemed incomplete, a fourth measurement was conducted. For the analysis, the mean systolic and diastolic blood pressure values were calculated from all available valid readings.

Participants were defined as having hypertension if they met any of the following criteria: self‐reported physician diagnosis of hypertension, current use of prescribed antihypertensive medication, or measured systolic blood pressure ≥ 140 mmHg and/or diastolic blood pressure ≥ 90 mmHg (Chobanian 2003).

### 2.3 Covariates

Potential covariates were selected based on a directed acyclic graph (DAG). Two adjusted models were constructed. Model I included age, gender, and race/ethnicity. Model II further included education levels, poverty income ratio (PIR), and smoking status and was specified as the primary model based on the DAG‐informed adjustment strategy. Additional variables available in the analytic dataset, including marital status, alcohol consumption, total energy intake from day 1 dietary recall, physical activity, oral hygiene behaviors, hyperlipidemia, diabetes mellitus, and frailty status, were not included in the primary model because they were not part of the main DAG‐informed adjustment set, and their inclusion might have resulted in overadjustment. Definitions of these additional variables are provided in Supporting Information S2. These variables were instead examined in sensitivity analyses to assess the robustness of the findings under alternative adjustment strategies. The DAG is presented as Figure S1.

Participants were categorized into four racial/ethnic groups: non‐Hispanic White, non‐Hispanic Black, Hispanic, and other races. Education levels were classified as less than high school, high school graduate, and more than high school. Smoking status was defined as never, former, or current smoking according to lifetime cigarette consumption and current smoking behavior (ALHarthi et al. 2019). Based on PIR, household income was categorized as low (PIR ≤ 1.3), middle (1.3 < PIR ≤ 3.5), and high (PIR > 3.5).

### 2.4 Statistical Analysis

All NHANES datasets were merged and managed using EmpowerStats version 2.0 and 5.2 (http://www.empowerstats.net/analysis). Continuous covariates are presented as mean ± standard deviation, and categorical variables as counts and proportions. Group differences were assessed using the chi‐square test for categorical variables and one‐way analyses of variance (ANOVA) for continuous variables.

Multivariable logistic regression models were used to examine the association between BRI and periodontitis, with results reported as odds ratios (OR) and 95% confidence intervals (CI). To explore the dose‐response pattern between BRI and periodontitis, smooth curve fitting was performed (He et al. 2025). The two‐piecewise regression model was applied to examine the potential threshold effect. The log‐likelihood ratio test was used to compare the two‐piecewise regression model with the one‐linear model (Li et al. 2022).

Stratified analyses were conducted across covariate‐defined subgroups. In sensitivity analyses without excluding participants with missing covariate data, missing covariate values were handled using multiple imputation to evaluate the potential impact of missingness. Additional sensitivity analyses were performed under alternative adjustment strategies. Specifically, panel A further adjusted for marital status, alcohol consumption, day 1 total energy intake, physical activity, and oral hygiene behaviors, including dental floss use; panel B additionally included hyperlipidemia and diabetes mellitus; and panel C further included frailty status. In addition, participants with mild periodontitis were excluded, and the association between BRI and moderate‐to‐severe periodontitis was re‐examined. A two‐sided *p* value < 0.05 was considered statistically significant.

## 3 Results

Table 1 shows that baseline characteristics differed significantly across the BRI quartiles. With increasing BRI, PIR generally decreased, the proportion of men decreased, and the proportion of women increased. Significant differences were also observed in age, race/ethnicity, education levels, and smoking status across the groups (all *p* < 0.05). Overall, baseline demographic and socioeconomic characteristics varied significantly across BRI quartiles.

**Table 1:** Descriptive characteristics of the study population stratified by BRI.

	Q1 (< 4.59)	Q2 (4.59–5.82)	Q3 (5.82–7.32)	Q4 (≥ 7.32)	
Characteristics	(*n* = 1029)	(*n* = 1029)	(*n* = 1029)	(*n* = 1030)	*p* value
Age (years)	58.03 ± 14.24	59.14 ± 13.64	59.34 ± 13.30	56.79 ± 13.12	< 0.001
PIR	2.71 ± 1.68	2.70 ± 1.66	2.54 ± 1.62	2.32 ± 1.51	< 0.001
Gender, *n* (%)					< 0.001
Male	600 (58.31)	616 (59.86)	508 (49.37)	383 (37.18)	
Female	429 (41.69)	413 (40.14)	521 (50.63)	647 (62.82)	
Race/ethnicity, *n* (%)					< 0.001
Hispanic	124 (12.05)	218 (21.19)	253 (24.59)	234 (22.72)	
Non‐Hispanic white	435 (42.27)	454 (44.12)	463 (45.00)	448 (43.50)	
Non‐Hispanic black	311 (30.22)	248 (24.10)	250 (24.30)	303 (29.42)	
Other race	159 (15.45)	109 (10.59)	63 (6.12)	45 (4.37)	
Education levels, *n* (%)					0.001
Less than high school	212 (20.60)	243 (23.62)	284 (27.60)	278 (26.99)	
High school	234 (22.74)	249 (24.20)	248 (24.10)	245 (23.79)	
More than high school	583 (56.66)	537 (52.19)	497 (48.30)	507 (49.22)	
Smoking status, *n* (%)					< 0.001
Never	530 (51.51)	523 (50.83)	546 (53.06)	566 (54.95)	
Former	260 (25.27)	348 (33.82)	313 (30.42)	305 (29.61)	
Now	239 (23.23)	158 (15.35)	170 (16.52)	159 (15.44)	

Abbreviations: BRI, body roundness index; PIR, poverty income ratio.

In the logistic regression analysis, no significant association between BRI and periodontitis was observed in the crude model (OR = 1.00,95% CI: 0.97–1.03, *p* = 0.9209). However, after adjustment for covariates in Model I and Model II, a statistically significant association between BRI and periodontitis was observed (*p* < 0.05). When BRI was analyzed by quartiles, participants in the highest quartile (Q4) had significantly higher odds of periodontitis than those in the lowest quartile (Q1) in both Model I (OR = 1.27, 95% CI: 1.05–1.54, *p* = 0.0122) and Model II (OR = 1.25, 95% CI: 1.03–1.53, *p* = 0.0258) (Table 2).

**Table 2:** The association between BRI and periodontitis.

	Crude Model		Model I		Model II	
Variable	OR (95% CI)	*p*	OR (95% CI)	*p*	OR (95% CI)	*p*
BRI (continuous)	1.00 (0.97, 1.03)	0.9209	1.04 (1.01, 1.07)	0.0106	1.04 (1.00, 1.07)	0.0260
BRI						
Quartile 1	Reference		Reference		Reference	
Quartile 2	1.07 (0.89, 1.27)	0.4732	1.02 (0.85, 1.23)	0.8229	1.06 (0.87, 1.29)	0.5672
Quartile 3	0.96 (0.80, 1.14)	0.6235	0.98 (0.81, 1.18)	0.8146	0.96 (0.79, 1.16)	0.6575
Quartile 4	1.04 (0.87, 1.24)	0.6411	1.27 (1.05, 1.54)	0.0122	1.25 (1.03, 1.53)	0.0258
*p* for trend	1.00 (0.95, 1.06)	0.9522	1.07 (1.01, 1.14)	0.0260	1.06 (1.00, 1.13)	0.0699

*Note:* Crude model adjust for: None. Model I adjust for: Age, gender, and race. Model II adjust for: Age, gender, race, education levels, PIR, and smoking status.

Abbreviations: BRI, body roundness index; CI, confidence interval; OR, odds ratio; PIR, poverty income ratio.

**Figure 2:**
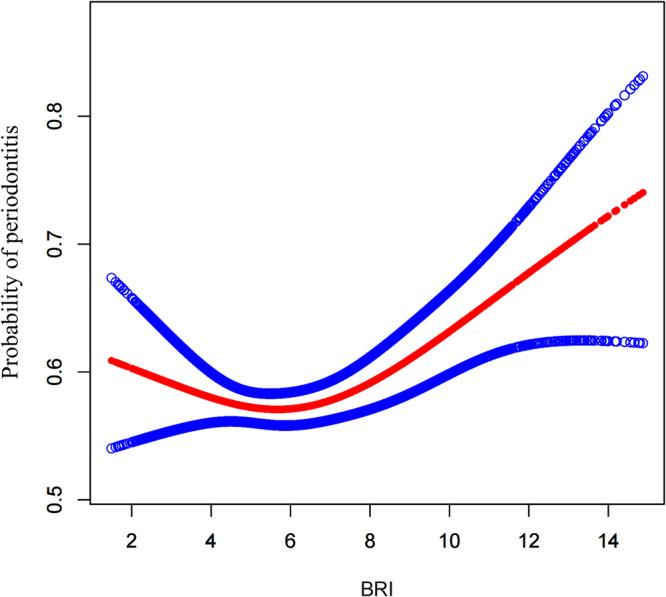
Dose‐response relationship between BRI and the probability of periodontitis. A threshold, nonlinear relationship between BRI and the probability of periodontitis was found in a generalized additive model. The solid red line represents the smooth curve fit between variables. Blue bands represent the 95% of confidence interval from the fit. All adjusted for age, gender, race, education levels, PIR, and smoking status. BRI, body roundness index; PIR, poverty income ratio.

Based on the regression analysis results according to BRI quartiles, we further applied a generalized additive model to perform smooth curve fitting. As shown in Figure 2, BRI appeared to have a nonlinear association with periodontitis.

As shown in Table 3, the log‐likelihood ratio test was statistically significant (*p* < 0.05). Based on visual inspection of the smoothed curve and consideration of the quartile‐based findings, the inflection point was estimated to be approximately 6. When BRI was below 6, the OR for the association between BRI and periodontitis was 0.94 (95% CI: 0.87–1.02, *p* = 0.1209), whereas when BRI was greater than or equal to 6, the corresponding OR was 1.09 (95% CI: 1.04–1.15, *p* = 0.0006).

**Table 3:** Threshold effect analysis of the BRI and periodontitis.

Models	Periodontitis	
	OR (95% CI)	*p* value
Model I		
One‐line effect	1.04 (1.00, 1.07)	0.0260
Model II		
Turning point (K)	6.00	
BRI < K	0.94 (0.87, 1.02)	0.1209
BRI ≥ K	1.09 (1.04, 1.15)	0.0006
Log‐likelihood ratio test		0.008

*Note:* Data were presented as OR (95% CI) *p* value; Model I, linear analysis; Model II, non‐linear analysis. Adjusted for age, gender, race, education levels, PIR, and smoking status.

Abbreviations: BRI, body roundness index; CI, confidence interval; OR, odds ratio; PIR, poverty income ratio.

**Figure 3:**
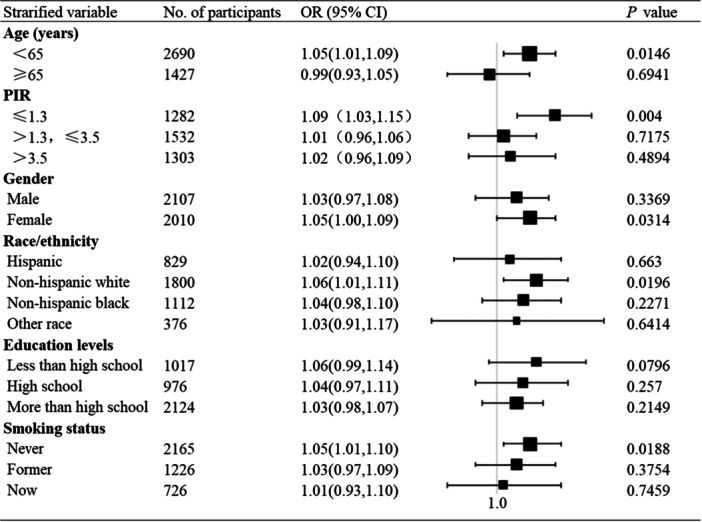
Stratified analyses of the association between BRI and periodontitis. BRI, body roundness index; PIR, poverty income ratio.

Stratified analyses were further performed according to age, PIR, gender, race/ethnicity, education levels, and smoking status. After adjustment for potential confounders, the association between BRI and periodontitis remained statistically significant among participants aged < 65 years, those with PIR ≤ 1.3, females, non‐Hispanic whites, and never smokers, whereas no significant association was observed in the other subgroups. Detailed results are presented in Figure 3.

Sensitivity analyses showed that the association between BRI and periodontitis was materially unchanged in the imputed dataset (Table 4). The nonlinear association remained robust after further adjustment for marital status, alcohol consumption, day 1 total energy intake, physical activity, and oral hygiene behaviors, including dental floss use (Figure S2A). The association was not materially altered after additional adjustment for hyperlipidemia and diabetes mellitus (Figure S2B), and further adjustment for frailty status also yielded consistent results (Figure S2C). In addition, after excluding participants with mild periodontitis, the association between BRI and moderate‐to‐severe periodontitis was consistent with that observed in the primary analysis including all periodontitis cases (Figure S3).

**Table 4:** Threshold effect analysis of the BRI and periodontitis among imputation data.

	Imputation 1	Imputation 2	Imputation 3	Imputation 4	Imputation 5
	OR (95% CI)	OR (95% CI)	OR (95% CI)	OR (95% CI)	OR (95% CI)
Model I					
One‐line effect	1.04 (1.01, 1.07)	1.04 (1.01, 1.07)	1.04 (1.01, 1.07)	1.04 (1.01, 1.07)	1.04 (1.01, 1.07)
Model II					
Turning point (K)	6.00	6.00	6.00	6.00	6.00
BRI < K	0.93 (0.86, 1.01)	0.93 (0.87, 1.01)	0.93 (0.87, 1.01)	0.93 (0.86, 1.00)	0.93 (0.86, 1.00)
BRI ≥ K	1.10 (1.05, 1.15)	1.10 (1.05, 1.15)	1.09 (1.05, 1.14)	1.09 (1.05, 1.14)	1.10 (1.05, 1.15)
Log‐likelihood ratio test	0.002	0.002	0.003	0.002	0.002

*Note:* Data were presented as OR (95% CI) *p* value; Model I, linear analysis; Model II, non‐linear analysis. All adjusted for age, gender, race, education levels, PIR, and smoking status.

Abbreviations: BRI, body roundness index; CI, confidence interval; OR, odds ratio; PIR, poverty income ratio.

## 4 Discussion

In this cross‐sectional study of US adults with hypertension using NHANES data from 2009 to 2014, BRI was associated with periodontitis after adjustment for DAG‐informed covariates. Participants in the highest BRI quartile had significantly higher odds of periodontitis than those in the lowest quartile. Smooth curve fitting and two‐piecewise regression analyses further suggested a nonlinear association between BRI and periodontitis. Sensitivity analyses using moderate‐to‐severe periodontitis and multiple imputation yielded broadly consistent results, supporting the robustness of the findings.

These findings add to the limited evidence linking BRI to periodontitis. Previous studies in general populations have reported positive associations between higher BRI and periodontitis (Xu and Fu 2025; Zhang et al. 2025). Our results extend this evidence to adults with hypertension and further suggest that the association was nonlinear. Therefore, BRI may provide additional information for periodontal risk assessment in this high‐risk population.

A notable finding was that the association appeared to be nonlinear rather than linear. Although the unadjusted linear model was not significant, significant associations emerged after adjustment, suggesting the potential influence of confounding by demographic, socioeconomic, and smoking‐related factors. The higher odds observed in the highest BRI quartile, along with the findings from smooth curve fitting and threshold‐effect analysis, further indicate that a single linear model may not adequately capture the association pattern across the full BRI range.

The positive association observed at or above the inflection point was generally consistent with previous findings in general populations and may be biologically plausible. Prior studies have shown that obesity is associated with chronic low‐grade inflammation (Grosso et al. 2022), altered adipokine secretion (Thanakun et al. 2017), and oxidative stress (Atabay et al. 2017), all of which have been proposed as potential mechanisms contributing to periodontal inflammation and tissue destruction (Reytor‐González et al. 2024). Obesity has also been linked to alterations in bone metabolism and osteoblast differentiation, which may plausibly contribute to periodontal tissue breakdown (Zhao et al. 2022). In addition, although some studies have suggested that obesity may be associated with changes in the periodontal microbiota (Haffajee and Socransky 2009; Maciel et al. 2016), the available evidence remains limited, and the causal contribution of such changes to periodontal bone loss is still unclear.

In contrast, below the inflection point, no statistically significant association between BRI and periodontitis was observed; therefore, this segment of the association should be interpreted cautiously and should not be overinterpreted.

It should also be noted that previous studies have shown that periodontitis is unevenly distributed across demographic and socioeconomic subgroups, including age, gender, race/ethnicity, education levels, poverty income ratio, and smoking status (Chen, Wang et al. 2025; Leite et al. 2018; Montero et al. 2019). These factors are also closely related to obesity and body fat distribution (Carrasquilla et al. 2024; Zare et al. 2021; Zheng et al. 2022). Accordingly, these variables were included in the primary model under the DAG‐informed framework. By contrast, other available variables, such as diabetes mellitus, hyperlipidemia, certain behavioral factors, and frailty, were examined in sensitivity analyses to assess the robustness of the findings under alternative adjustment strategies.

We also conducted stratified analyses. Although statistically significant associations were observed only in some subgroups, the overall pattern of effect estimates in the remaining subgroups was broadly consistent with that of the primary analysis. Given the exploratory nature of these analyses and the reduced precision after subgroup stratification, they should not be overinterpreted as evidence of true subgroup‐specific heterogeneity.

We used the CDC/AAP case definition because it was developed for population‐based surveillance and is widely used in studies based on NHANES data (Eke et al. 2012). Although the 2017 World Workshop classification is the current clinical standard, its full implementation in NHANES is limited by the lack of key information, including radiographic bone loss, tooth loss attributable to periodontitis, complexity‐related factors, and disease progression data, as well as the inability to fully exclude attachment loss due to non‐periodontitis causes (Papapanou et al. 2018). Therefore, the CDC/AAP case definition was considered more appropriate for the present analysis based on NHANES data.

Several limitations should be noted. First, the cross‐sectional design precludes conclusions about temporality or causality. Second, although the association between BRI and periodontitis was statistically significant, the magnitude of the association was relatively small; therefore, the finding should be interpreted with caution. Third, the study population was restricted to adults with hypertension; therefore, the findings may not be directly generalizable to individuals without hypertension. Fourth, although covariate selection was guided by the DAG and the findings were broadly consistent after additional adjustment for variables such as frailty, hyperlipidemia, and diabetes mellitus, residual confounding from unmeasured or incompletely measured factors remains possible. Lower BRI may reflect health heterogeneity beyond adiposity, which may not have been fully captured by the available covariates. Fifth, use of the CDC/AAP definition may limit direct comparability with studies based on the 2017 classification.

Overall, our findings suggest a nonlinear association between BRI and periodontitis among US adults with hypertension. Prospective studies are needed to confirm these findings and clarify the underlying mechanisms.

## 5 Conclusions

In this cross‐sectional study of US adults with hypertension, BRI was associated with periodontitis, and the association was nonlinear. Further large‐scale longitudinal studies with robust study designs are needed to validate these findings and clarify the mechanisms underlying the observed association.

## Author Contributions

Liting Xie conceived and designed the study, performed comprehensive data analysis and interpretation, drafted the manuscript, and made substantial revisions. Xiaobin Zhang contributed to the study conception and methodological design and critically revised the manuscript. Zhiyong Pan and Zhiqiang Xu contributed to the study conception, refined the research design, and contributed to manuscript revisions. All authors reviewed and approved the final version of the manuscript. Zhiyong Pan and Zhiqiang Xu are co‐corresponding authors. Zhiyong Pan is the primary contact for correspondence.

## Ethics Statement

All data utilized in this study were derived from NHANES. The NHANES study protocols were reviewed and approved by the NCHS Institutional Review Board. All procedures complied with the ethical standards stipulated in the Declaration of Helsinki. Since the dataset used in the present analysis is de‐identified and publicly available, additional Institutional Review Board approval for this secondary analysis was not required.

## Consent

Written informed consent was obtained from all participants prior to data collection and health examinations. All authors have agreed to the publication of this manuscript.

## Conflicts of Interest

The authors declare no conflicts of interest.

## Supporting information


Supporting File 1

Supporting File 2

## Data Availability

The NHANES dataset is publicly available online, accessible at https://www.cdc.gov/nchs/nhanes/.
