# Supplementary material for: Association Between Body Roundness Index and Periodontitis in Adults With Hypertension: A Cross‐Sectional Study
Source: Clin Exp Dent Res. 2026 Aug 13;12(4):e70429. doi: 10.1002/cre2.70429 (PMC13472514; doi:10.1002/cre2.70429)
Supplement: Supplementary file 1 [file CRE2-12-e70429-s001.docx]

**Definitions of additional variables used in the sensitivity analyses**

Marital status was defined as married/living with a partner, widowed/divorced/separated, or never married. Alcohol consumption was classified as never, former, and current drinkers. Physical activity was grouped as inactive (0 min/week), insufficiently active (1-149 min/week), and recommended (≥150 min/week). Day 1 total energy intake was obtained from the 24-hour dietary recall file (DR1TKCAL). Hyperlipidemia and diabetes mellitus were defined according to laboratory criteria, self-reported physician diagnosis, or relevant medication use (Pu et al., 2025; Yao et al., 2024). Oral hygiene behaviors were categorized into low (0–1 days/week), moderate (2–4 days/week), and high frequency (≥5 days/week) according to the frequency of dental flossing and use of other dental cleaning devices. Frailty was assessed using a 49-item deficit accumulation frailty index covering seven domains, calculated as the proportion of deficits present among participants with at least 80% item completion, with FI＞0.21 indicating frailty (Yan, Chai, Yang, & Wang, 2024).

**References**

Pu, K., Luo, T., Li, J., Tang, Q., Feng, Y., & Yang, G. (2025). Periodontitis and gastrointestinal cancer: a nationwide cohort study of NHANES 2009-2014. *BMC Public Health, 25*(1), 804. doi:10.1186/s12889-025-21832-2

Yan, S., Chai, K., Yang, J., & Wang, H. (2024). Association of visceral adiposity index and lipid accumulation product with frailty in U.S. adults: a cross-sectional study from NHANES. *Lipids Health Dis, 23*(1), 417. doi:10.1186/s12944-024-02410-8

Yao, Y., Wang, B., Geng, T., Chen, J., Chen, W., & Li, L. (2024). The association between TyG and all-cause/non-cardiovascular mortality in general patients with type 2 diabetes mellitus is modified by age: results from the cohort study of NHANES 1999-2018. *Cardiovasc Diabetol, 23*(1), 43. doi:10.1186/s12933-024-02120-6
