# Supplementary material for: Association Between Body Roundness Index and Periodontitis in Adults With Hypertension: A Cross‐Sectional Study
Source: Clin Exp Dent Res. 2026 Aug 13;12(4):e70429. doi: 10.1002/cre2.70429 (PMC13472514; doi:10.1002/cre2.70429)
Supplement: Supplementary file 2 [file CRE2-12-e70429-s002.docx]

Fig.S1. Directed acyclic graph (DAG) showing the presumed causal relationships among BRI, periodontitis, and selected covariates. The DAG was used to identify the minimally sufficient adjustment set for the primary analysis. Red, green, and blue ovals represent variables that are ancestors of both BRI and periodontitis, BRI only, and periodontitis only, respectively; gray nodes represent other variables.

Abbreviations: BRI, body roundness index; PIR, poverty income ratio

Fig.S2. Dose-response relationships between BRI and periodontitis.

The solid red line represents the smoothed fitted curve, and the blue brand represents the 95% confidence interval. (A) Further adjusted for marital status, alcohol consumption, frequency of dental flossing, day 1 total energy intake, and physical activity in addition to the covariates included in the primary model. (B) Further adjusted for hyperlipidemia and diabetes mellitus in addition to the variables included in panel A. (C) Further adjusted for frailty status in addition to the variables in panel B.

Abbreviations: BRI, body roundness index; PIR, poverty income ratio.

Fig.S3. Dose-response relationship between BRI and the probability of moderate-to-severe periodontitis

Solid red line represents the smooth curve fit between variables. Blue bands represent the 95% of confidence interval from the fit.

All adjusted for age, gender, race, education levels, PIR, smoking status.

Abbreviations: BRI, body roundness index; PIR, poverty income ratio.
